# The pituitary tumor transforming gene 1 (PTTG-1): An immunological target for multiple myeloma

**DOI:** 10.1186/1479-5876-6-15

**Published:** 2008-04-02

**Authors:** Maurizio Chiriva-Internati, Raffael Ferraro, Madhavi Prabhakar, Yuefei Yu, Luigi Baggoni, Jorge Moreno, Nicoletta Gagliano, Nicola Portinaro, Marjorie R Jenkins, Eldo E Frezza, Fred Hardwicke, Nicholas D'Cunha, WMartin Kast, Everardo Cobos

**Affiliations:** 1 Department of Microbiology and Immunology, Texas Tech University Health Sciences Center and Southwest Cancer Treatment and Research Center, Lubbock, TX, USA; 2 Division of Hematology and Oncology, Texas Tech University Health Sciences Center and Southwest Cancer Treatment and Research Center, Lubbock, TX, USA; 3 Division of Surgery, Texas Tech University Health Sciences Center and Southwest Texas Tech University Health Sciences Center and Southwest Cancer Treatment and Research Center, Lubbock, TX, USA; 4 Department of Human Morphology, University of Milan, Milan, Italy; 5 Department of Internal Medicine, Obstetrics & Gynecology and Laura W. Bush Institute for Women's Health and Center for Women's Health and Gender-Based Medicine, Texas Tech University Health Sciences Center, Amarillo, TX, USA; 6 Department of Pediatric Orthopedic Surgery, Istituto Clinico Humanitas, Rozzano, Milan, Italy; 7 Department of Molecular Microbiology & Immunology and Obstetrics & Gynecology, Norris Comprehensive Cancer Center, University of Southern California, Los Angeles, CA, USA

## Abstract

**Background:**

Multiple Myeloma is a cancer of B plasma cells, which produce non-specific antibodies and proliferate uncontrolled. Due to the potential relapse and non-specificity of current treatments, immunotherapy promises to be more specific and may induce long-term immunity in patients. The pituitary tumor transforming gene 1 (PTTG-1) has been shown to be a novel oncogene, expressed in the testis, thymus, colon, lung and placenta (undetectable in most other tissues). Furthermore, it is over expressed in many tumors such as the pituitary adenoma, breast, gastrointestinal cancers, leukemia, lymphoma, and lung cancer and it seems to be associated with tumorigenesis, angiogenesis and cancer progression. The purpose was to investigate the presence/rate of expression of PTTG-1 in multiple myeloma patients.

**Methods:**

We analyzed the PTTG-1 expression at the transcriptional and the protein level, by PCR, immunocytochemical methods, Dot-blot and ELISA performed on patient's sera in 19 multiple myeloma patients, 6 different multiple myeloma cell lines and in normal human tissue.

**Results:**

We did not find PTTG-1 presence in the normal human tissue panel, but PTTG-1 mRNA was detectable in 12 of the 19 patients, giving evidence of a 63% rate of expression (data confirmed by ELISA). Four of the 6 investigated cell lines (66.6%) were positive for PTTG-1. Investigations of protein expression gave evidence of 26.3% cytoplasmic expression and 16% surface expression in the plasma cells of multiple myeloma patients. Protein presence was also confirmed by Dot-blot in both cell lines and patients.

**Conclusion:**

We established PTTG-1's presence at both the transcriptional and protein levels. These data suggest that PTTG-1 is aberrantly expressed in multiple myeloma plasma cells, is highly immunogenic and is a suitable target for immunotherapy of multiple myeloma.

## Background

Multiple myeloma (MM) is a malignancy of B plasma cells (PCs). They accumulate in the bone marrow (BM) causing bone destruction, BM failure [1] and interfere with the normal PC activity by generating an abnormal, non-functional and non-specific immunoglobulin (M protein) [2]. Further, the malignant plasma cells can also be found in extra-medullary locations, such as peripheral blood, pleural effusion and ascites [3]. Every year about 15,000 new cases of MM are diagnosed in the U.S. [1]. The median age of diagnosis is 67 years (it rarely occurs before age 45) and the median survival is 3 to 4 years [4]. MM onset is still not understood and since relapses are systematically observed after transient complete remission, it remains an incurable hematological disease [5]. Treatment for MM is currently based on high doses of chemotherapy, radiotherapy and autologous stem cell rescue, but death is the ultimate outcome.

Since the identification of tumor-associated antigens (TAA), capable of inducing an immune response in cancer patients, has become a formidable task for tumor immunologists [6], the research of new possible target candidates holds high promise for the success of biological therapy [7,8]. In fact, the immune system is capable of discriminating between benign and malignant cells by recognizing aberrantly expressed proteins/peptides exposed on the cell surface in the context of the major histocompatibility complex (MHC) [9]. Immune responses are also highly dependant on the tumor's micro-environment [10]. It has been shown that the addition of systemic Interleukin-2 (IL-2) therapy to tumor immunization plays a pivotal role in increasing the frequency of immune cancer rejections [10]. The identification of novel TAA is only the first step in improving the complementary use of two biotherapic approaches (active immunization/adoptive transfer of tumor antigen-specific T cells) and to better design future simple and safe clinical studies [11].

In the last decade, PTTG-1 has been shown to be a novel oncogene [12]. Human PTTG-1 is located on chromosome 5 and encodes a protein of 202 amino acids (22 kDa) [13]. PTTG-1 is involved in transcriptional and cell cycle regulation with expression in the normal testis and thymus, and weak expression signals in colon, small intestine, brain, placenta, and pancreas [12]. Further investigations showed PTTG-1 to be highly expressed in different tumor cell lines (promyelocytic leukemia cell line HL-60, HeLa cell S3, chronic myelogenous leukemia cell line K-562, lymphoblastic leukemia cell line MOLT-4, Burkitt's lymphoma cell line Raji, colorectal adenocarcinoma cell line SW480, lung carcinoma cell A549, melanoma cell G361) [12]. PTTG-1 has also been shown to be tumorigenic *in vivo *[14] and, it seems to be associated with tumorigenesis, angiogenesis and cancer progression [12]. In tumorigenesis, PTTG-1 might be playing a dual role. First, over-expression of PTTG-1 initiates genetic instability and, second, high PTTG-1 expression induces the transduction of fibroblast growth factor 2 (FGF-2), vascular endothelial growth factor (VEGF) and other pro-angiogenic genes [15]. Investigated in tumors, PTTG-1 has been found over expressed in pituitary tumors, thyroid cancer [16,17], esophageal squamous cancer [18,19], uterine leioma [20], lung cancer [21,22], lymphoid cancer [23,24], colon cancer [25], gastric carcinoma [26] testicular cancer [27], breast cancer [28], astrocytoma cancer [29,30].

Recently PTTG-1 was investigated in MM and found to be expressed at the transcriptional level [31]. We wanted to further investigate MM cases evaluating both the mRNA and the protein level, comparing our results to a human normal tissue panel in order to give room to the hypothesis of using PTTG-1 as a target for biological therapy in MM.

## Methods

### Patients and materials

We evaluated human normal tissues by means of a normal tissue panel (Applied Biosystems, Foster City, CA, USA) and a normal tissue panel array (Pantomics, San Francisco, CA, USA) prepared for brain, breast, colon, heart, kidney, liver, lung, ovary, pancreas, skeletal muscle, spleen, stomach, trachea and bone marrow. Plasma cells of 19 MM patients (purified by BB4 antibody) and 6 established MM cell lines (KMS11, 8226, ARK-B, ARP-1, U266, OPM2) were investigated. All of the clinical materials were obtained with the patient consent and approval from the local ethics committee. PTTG-1 expression was evaluated by Reverse Transcription-Polymerase Chain Reaction (RT-PCR), immunohisto/cytochemistry (IHC/ICC), immunofluorescence (IF) and fluorescence activated cell sorter (FACS).

### RT-PCR

PCR analysis has been performed as previously described [32,33]. Briefly, 1 μg of total RNA extracted from cells by Tri-reagent (Sigma, St Louis, MO, USA) was DNAse I digested (Ambion, Austin, TX, USA) and reverse-transcribed by random hexamers. The primers sequences were as follows: 5'-GGT TTA AAC CAG GAG TGC CGC-3' and 5'-AAT TCA ACA TCC AGG GTC GAC AG-3' (35 cycles, annealing 55°C). RNA integrity in each sample was checked on β-actin gene expression. All results were confirmed in 3 independent RT-PCRs.

### Immunohistochemistry

Experiments were performed as previously described [33]. We used anti-PTTG-1 (Zymed Laboratories, San Francisco, CA, USA) as primary antibody at a dilution factor of 1:100 (diluted in PBS 1× + BSA 0.1%). The DAKO HRP-labeled Envision system was used as secondary antibody (DAKO, Carpinteria, CA, USA), followed by 5 minutes dark incubation with DAB system (DAKO), used to yield brown reaction products. Cells were counter-stained with hematoxylin (Fisher Scientific, Pittsburg, PA, USA) and results were evaluated by light microscope (Leica DMLA, USA). Pictures were taken at 20×, 40× and 63× ranges of magnitude and analyzed by Isole software.

### Immunocytochemistry/Immunofluorescence

Experiments were performed as previously described [33]. MM plasma cells and cell lines were spun in a cytospin column (5 × 10^4 ^cells/slide), fixed with SlideRite (Fisher, USA) and air-dried overnight. Each sample was either permeabilized (P) in PBS1X/0.1% Triton X-100 for 15 minutes at 4°C or not permeabilized (NP). For ICC, cells were treated with anti-PTTG-1 primary antibody (Zymed Lab) (1:100 dilution), incubated for 30 minutes with the Envision System (DAKO) and 5 minutes with DAB (DAKO). The ICC reaction was observed by light microscope (Leica). For IF, cells were incubated overnight in a wet chamber at 4°C with anti-PTTG-1 primary antibody (Zymed Lab) (1:100 dilution), then with FITC conjugated IgG secondary antibodies (1:500, Abcam, USA). Results were analyzed using an Olympus IX71 inverted microscope equipped with a Fluoview 300 confocal laser system.

### Protein generation

**Figure 1:**
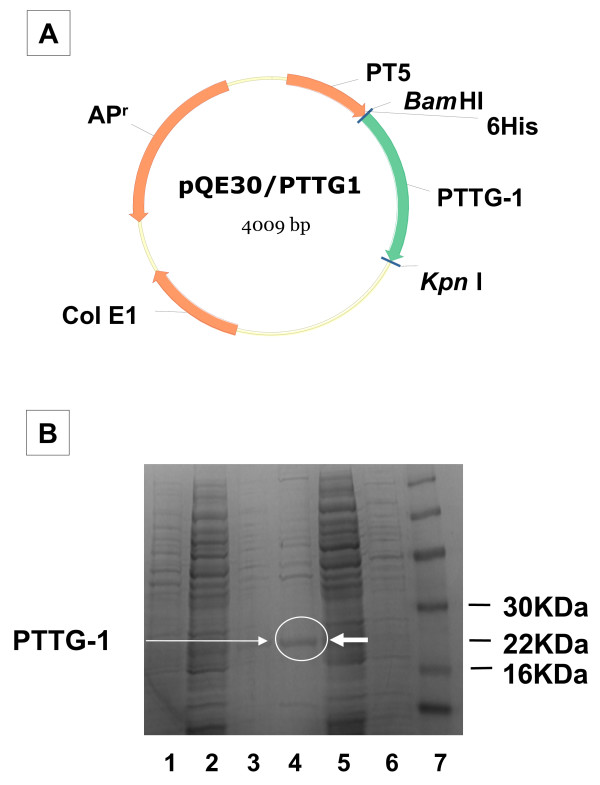
**1A**. **Scheme of pQE30 plasmid for the generation of PTTG-1 protein.1B. **SDS-PAGE gel for purification of PTTG-1 protein stained with commassie blue. Line 1) washing buffer, 2) flow through fraction, 3) elution 2, 4) elution 1, 5) pQE30/pttg-1 with IPTG, 6) pQE30/pttg-1 without IPTG, 7) Marker.

PTTG-1 protein was generated through the use of PQE30 plasmid (Fig. 1A) transformed into M15 *E. coli *cells. This plasmid contains ampicillin resistance gene and 6× His-tag. IPTG (1 μM) was added as a promoter inducer once the cultures were grown and an O.D. of 0.6 was reached. Following growth of the *E. coli *cells, Qiagen Ni-NTA Fast Start Kit was used from the cell lysis step till the purification step by the nickel columns provided in the kit. SDS-PAGE (10%) analysis confirmed the purification of the PTTG-1 (final concentration 5 mg/ml) protein (Fig. 1B).

### Enzyme-linked immunosorbent assay

An enzyme-linked immunosorbent assay (ELISA) was performed [33] on the sera of 19 MM patients and 11 healthy donors with no known abnormalities. Polystyrene 96-well flat-bottom plates were coated with PTTG-1 recombinant protein (5 μg/μl) and incubated overnight at 4°C. After washing and blocking with SuperBlock^® ^buffer (Pierce, Rockford, IL, USA), plates were placed at 37°C for 2 hours. Each sample, as well as the negative controls (PBS/FBS 1×) was diluted 1:1000 in SuperBlock^® ^buffer and incubated for 4 hours at RT. After washing with PBS/Tween20 0.05%, horseradish peroxidase conjugated goat anti-human IgG (Pierce), diluted 1:5000 in SuperBlock^®^, was added and allowed to incubate at RT for 2 hours. Then 1-Step Ultra TMB-ELISA chromogenic substrate (Pierce) was added to each well for color development for 10 minutes. After blocking the reaction with sulfuric acid, the intensity was measured by Victor 2 micro plate multilabel counter (PerkinElmer, Waltham, MA, USA) at 450 nm. All samples were run in triplicates.

### Dot-blot

The presence of PTTG-1 protein was also evaluated by Dot-blot. Cell lysates of 5 MM cell lines and 19 MM patients were blotted on a nitrocellulose membrane by vacuum aspiration in a Bio-Dot Apparatus (Bio-Rad Laboratories). The membrane dried at room temperature for 30 minutes and then blocked for 45 minutes in milk 5% and TBST (Tris 100 mM, NaCl 1.5 M, Tween20 0.05%). Membrane was washed in TBST 2 times and incubated for 1 h with the primary antibody (Zymed Lab) diluted 1:250. After 2 washes of 5 minutes in TBST, membrane was incubated for 1 hour with secondary antibody (ImmunoPure, PIERCE) diluted 1:5000. Immuno-revelation was performed incubating the membrane with Opti-4CN and Amplified Opti-4CN Kit (Bio-Rad Laboratories) for 15 min. All the samples were run in three different dilutions in TBS 1× (1:100, 1:5000, and 1:1000).

### FACS

The expression of PTTG-1 has been demonstrated by FACS analysis as previously described with a double stain reaction [32]. Briefly MM plasma cells and MM cell lines were incubated with anti-PTTG-1 primary antibody (Zymed Lab) diluted in PBS 1×. PBS 1× alone was used as negative control. PE-conjugated anti-rabbit IgG (Imgenex, San Diego, CA, USA) was used to detect and bind the primary antibody. Analysis was performed using a fluorescence-activated cell scanner (B&D, Bioscience-PharMingen, Franklin Lakes, NJ, USA).

## Results

**Figure 2:**
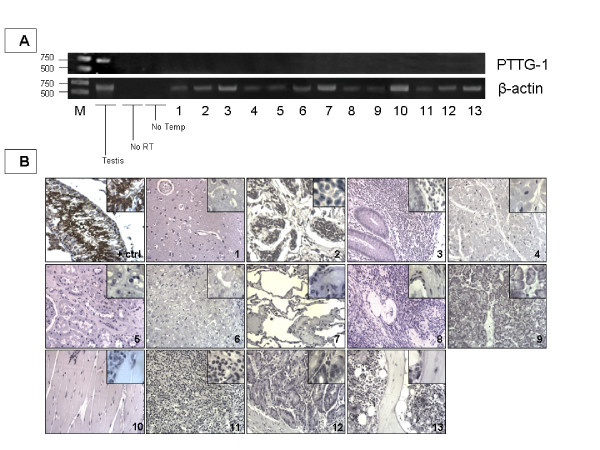
**2A. The PCR results of the normal tissue panel are shown.** Except for the positive control (the testis), none of the analyzed organs showed positive band signals. **2B. **IHC results did not show the presence of PTTG-1 at the protein level in the normal tissue array. Investigated tissues: 1) brain, 2) breast, 3) colon, 4) heart, 5) kidney, 6) liver, 7) lung, 8) ovary, 9) pancreas, 10) skeletal muscle, 11) spleen, 12) stomach and 13) bone marrow.

We wanted to investigate a panel of normal human tissues constituted by brain, breast, colon, heart, kidney, liver, lung, ovary, pancreas, skeletal muscle, spleen, stomach and bone marrow to evaluate the presence of PTTG-1 at both the transcriptional and the protein levels. Our investigation of the 13 above mentioned human normal tissues gave no evidence of positive band signals for PTTG-1 (Fig. 2A) by PCR. Also, the human normal tissue array, investigated by IHC, did not show positive staining (Fig. 2B), as expected and suggested by the PCR data.

**Figure 3:**
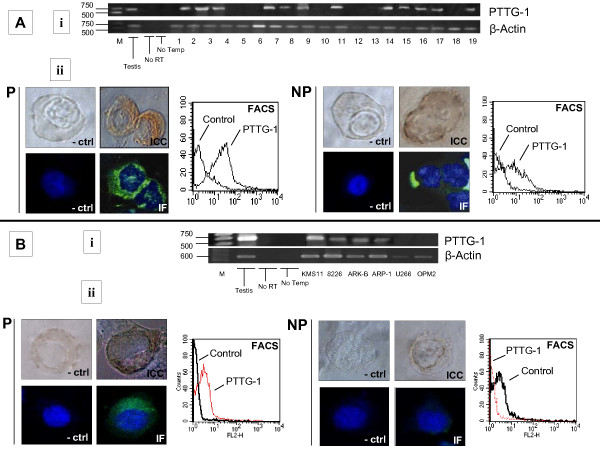
**3A. i. The PCR result shows 12/19 positive cases for PTTG-1 at the transcriptional level.ii. **Representative cases of patient MM plasma cells. In the (**P**) panel, the cytoplasmic staining is shown by ICC, IF and FACS. Also the **NP **panel shows a positive result for surface staining by ICC, IF and FACS methods. **3B. i. **The investigation of the 6 established MM cell lines gave positive band signals in the four cases of KMS-11, 8226, ARK-B and ARP-1. **ii**. The evaluation of PTTG-1 protein expression showed only cytoplasmic but not surface positive reaction. The case of the permeabilized (**P**) and not permeabilized (**NP**) 8226 cell line is shown by means of ICC, IF and FACS.

**Figure 4:**
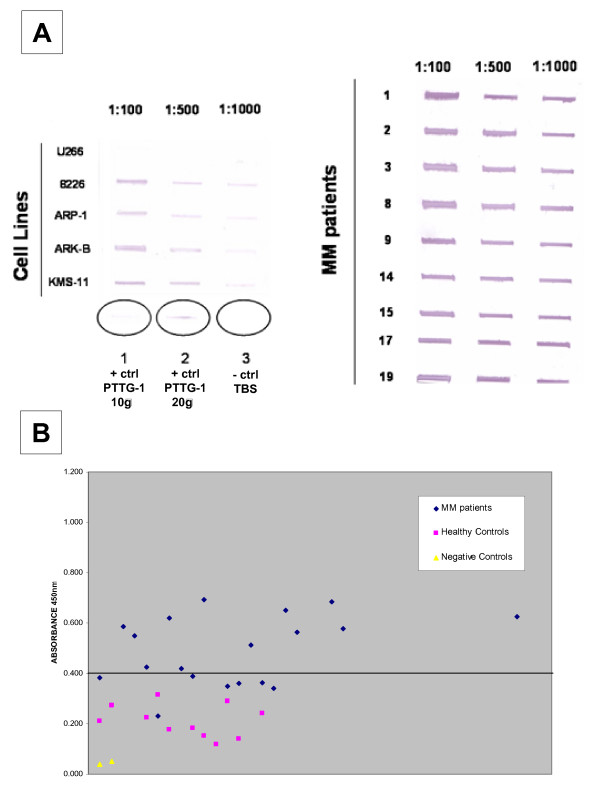
**A. Dot-blot confirms the PCR data in representative MM cell lines on the left (U266, 8226, ARP-1, ARK-B and KMS-11) and patients (negative patients not shown) on the right, in three different serial dilutions (1:100, 1:500, 1:1000).** 1) positive ctrl (PTTG-1 20 μg), 2) positive ctrl (PTTG-1 10 μg), 3) negative ctrl (TBS only). The primary antibody was diluted 1:250 (Zymed Lab), secondary antibody 1:5000 (Pierce). **B. **ELISA technique shows the presence of IgG against PTTG-1 in 12/19 MM patients (63%). The cut-off point (mean + 3 STDEV), based on 11 healthy controls' values, is OD_450 nm _= 0.404.

The evaluation of the plasma cells of 19 MM bearing patients gave evidence of 12 positive cases (Fig. 3Ai), showing a 63% expression rate. The further evaluation of 6 MM established cell lines, by PCR, showed 4 positive cases (67%): KMS-11, 8226, ARK-B and ARP-1 (Fig. 3Bi). We further investigated the MM plasma cells and the MM cell lines for the protein expression of PTTG-1. We wanted to specifically evaluate if PTTG-1 is expressed or shown at both the cytoplasmic and/or surface levels. Permeabilized and non-permeabilized cells were treated either by ICC or IF, and analyzed by microscope and further evaluated by FACS. The cytoplasmic expression of PTTG-1 was found in five of the 19 patients (26.3%), while the surface staining was detectable in three of the 19 patients (16%). Representative cases of cytoplasmic and surface protein expression are shown in Fig. 3Aii. In the MM cell lines, we found cytoplasmic staining only within the 4 positive MM cell lines, as shown in the representative case of the 8226 cell line (Fig. 3Bii). Protein presence was also confirmed by Dot-blot analysis, performed on MM cell lines lysates and patients sera (Fig. 4A).

To determine immunogenicity we investigated the presence of IgG antibodies against PTTG-1 in the serum of the 19 MM patients by ELISA (Fig. 4B). A positive signal was shown in 12/19 of the analyzed cases (63%). The cut-off point (mean + 3 STDEV), determined on the healthy controls, was significantly low with an OD_450 nm _= 0.4041, while negative controls had an OD_425 nm _= 0.044.

## Discussion

We report for the first time the interesting finding that PTTG-1 is aberrantly expressed in MM at both the transcriptional and the protein levels. Our investigations gave evidence of its presence, at the transcriptional level, in 63% of the analyzed cases. In our data, it was shown that PTTG-1 was not detectable at the mRNA or protein levels in human normal tissue panels, while other reports showed PTTG-1 to be weakly expressed in normal colon, lung, thymus, and placenta [12]. Since PTTG-1 has been described as an oncogene involved in tumorigenesis, its aberrant expression in MM may play a significant role in the onset and development of the disease.

A previous study investigated the presence of PTTG-1 in a pool of MM patients showing a significant over-expression of the gene at the transcriptional level [31]. Starting from this observation, and considering PTTG-1 as associated with MM, we wanted to extend the scenario analyzing the expression of PTTG-1 in MM PCs, specifically at the protein level.

Our data shows that PTTG-1 is present at the protein level both in the cytoplasmic district and, interestingly, on the surface of MM PCs. This is relevant because most of the tumor-associated antigens (TAAs) are not expressed on the surface of tumor cells, shadowing their usefulness as reliable targets, able to elicit an effective response of the host immune system [34].

ELISA results showed that PTTG-1 is immunogenic since anti-PTTG-1 IgG antibodies were found in 12/19 patients (63%). We suggest to conduct further studies in order to examine the machinery and the mechanisms involved in the protein's expression on the surface of MM PCs.

In fact, it has been shown that, in tumors, some proteins are present either in their native form or in a modified form (due to a mutation in the gene or to post-translational modifications) [34]. Modified and aberrantly expressed proteins can be processed in the tumor cell (by the proteasome) where they are reduced into small peptides of 8–15 AA [35] and then they are bound to MHC class I molecules in the endoplasmic reticulum [34]. The processed epitope is transported to the cell surface where it can induce an immune response of host CD8^+ ^CTLs [34,36]. In order to improve the specificity of antigen specific-CTL, there is ongoing research (based on algorithms) of highly immunogenic epitopes within TAAs [37]. Emphasis should also be placed on increasing our knowledge of cancer immunobiology, as well as on the improvement of cellular immune function monitoring, after vaccination [38]. Obstacles to effective translational medicine still remain [39]. For example, both the challenge of translating basic science discoveries into clinical studies and the translation of clinical studies into medical practice should be stressed [39]. In fact, high morbidity and mortality are attributable to limited current therapies; there is a need for a new generation of vaccines that are cost effective, safe and able to induce durable immune responses [40].

PTTG-1 has been described, in normal conditions, as a transcriptional factor, mainly expressed in the nuclei of cells. This gives room to the hypothesis that PTTG-1 may be expressed, in tumors, in a mutated form either acquiring a trans-membrane domain or getting processed by the above described machinery system. Nevertheless, in the case of modified, aberrantly expressed proteins, it is relevant to consider that post-translational modifications of the antigen may limit the cloning of effective CTLs. In fact these modifications need to be taken in consideration in order to clone highly specific CTLs and to improve the effectiveness of a CTL based biological therapy [34].

## Conclusion

Our findings suggest that PTTG-1 is a potential suitable target in MM. Even if further studies are needed to support this suggestion, there is a high probability that PTTG-1 is part of the rising group of new targets in MM whose discovery gives hope for the development, in the near future, of a successful treatment for MM, based on a polyvalent vaccine strategy [7].

## Competing interests

The author(s) declare that they have no competing interests.

## Authors' contributions

MCI carried out the study design, FACS analysis and drafted the manuscript and revised the manuscript. RF performed immunohistochemistry, immunocytochemistry, immunofluorescence experiments and drafted the manuscript. MP performed immunohistochemistry experiments. YY performed protein generation and all PCR experiments. LB performed Dot blot experiments. JM performed the ELISA experiment. NG participated in the design of the study and revised the manuscript. NP participated in the design of the study and revised the manuscript. MJ participated in study design and coordination and revised the manuscript. EF participated in the design of the study and revised the manuscript. FH participated in study design and coordination. ND participated in study design and coordination. WMK participated in study design and coordination and revised the manuscript. EC participated in study design and coordination and revised the manuscript. All authors read and approved the final manuscript.
